# A BERT model generates diagnostically relevant semantic embeddings from pathology synopses with active learning

**DOI:** 10.1038/s43856-021-00008-0

**Published:** 2021-07-05

**Authors:** Youqing Mu, Hamid R. Tizhoosh, Rohollah Moosavi Tayebi, Catherine Ross, Monalisa Sur, Brian Leber, Clinton J. V. Campbell

**Affiliations:** 1 McMaster University, Hamilton, ON Canada; 2 Kimia Lab, University of Waterloo, Waterloo, ON Canada; 3 Juravinski Hospital and Cancer Centre, Hamilton, ON Canada

**Keywords:** Pathology, Haematological cancer

## Abstract

**Background:**

Pathology synopses consist of semi-structured or unstructured text summarizing visual information by observing human tissue. Experts write and interpret these synopses with high domain-specific knowledge to extract tissue semantics and formulate a diagnosis in the context of ancillary testing and clinical information. The limited number of specialists available to interpret pathology synopses restricts the utility of the inherent information. Deep learning offers a tool for information extraction and automatic feature generation from complex datasets.

**Methods:**

Using an active learning approach, we developed a set of semantic labels for bone marrow aspirate pathology synopses. We then trained a transformer-based deep-learning model to map these synopses to one or more semantic labels, and extracted learned embeddings (i.e., meaningful attributes) from the model’s hidden layer.

**Results:**

Here we demonstrate that with a small amount of training data, a transformer-based natural language model can extract embeddings from pathology synopses that capture diagnostically relevant information. On average, these embeddings can be used to generate semantic labels mapping patients to probable diagnostic groups with a micro-average F1 score of 0.779 Â ± 0.025.

**Conclusions:**

We provide a generalizable deep learning model and approach to unlock the semantic information inherent in pathology synopses toward improved diagnostics, biodiscovery and AI-assisted computational pathology.

## Plain Language Summary

Pathology synopses are short texts describing microscopic features of human tissue. Medical experts use their knowledge to understand these synopses and formulate a diagnosis in the context of other clinical information. However, this takes time and there are a limited number of specialists available to interpret pathology synopses. A type of artificial intelligence (AI) called deep learning provides a possible means of extracting information from unstructured or semi-structured data such as pathology synopses. Here we use deep learning to extract diagnostically relevant textual information from pathology synopses. We show our approach can then map this textual information to one or more diagnostic keywords. We provide a generally applicable and scalable method to unlock the knowledge in pathology synopses as a step toward exploiting computer-aided pathology in the clinic.

## Abstract (web summary)

Mu et al. utilize a deep learning natural language processing model as part of an active learning approach to extract diagnostically relevant semantic information from bone marrow pathology synopses. Their findings demonstrate the potential for artificial intelligence in assisting clinicians in assessing, cataloging and triaging medical text datasets such as pathology synopses.

## Introduction

Making a diagnosis in pathology is a complex intellectual process, involving the integration of information from multiple pathological and clinical sources^1^. The pathologist’s central role is to extract visual information from microscopic features of human tissue (morphology), thereby lowering the uncertainty about a suspected disease state^2^. This information is then transferred into a written pathology report, which is synthesized in the context of the inherent world model and the knowledge accrued by the pathologist over many years. Therefore, a pathology report is intrinsic semantics of tissue morphology, which then must be captured and interpreted by an expert reader in the context of their world model and domain-specific knowledge. This requires years of specialized training, as pathologists often do not make a specific diagnostic interpretation^3^. Rather, a diagnosis often consists of semantic information extracted from the pathology specimen, ancillary testing, and the clinical history described as either unstructured or semi-structured text (called a synopsis). A pathology synopsis may give one or more probable diagnoses (i.e., a differential diagnosis) or may simply describe the salient morphological information without a differential diagnosis, and it is left to the expert end-reader to extract the semantic content. The reader must then map this semantic content to one of a small number of core concepts that help decide the appropriate next steps and diagnosis. This poses a challenge for knowledge mining given the finite number of experts who can do this, specifically when scaled to a large number of synopses. Tools to automatically extract the morphological semantics from pathology synopses would have high value in both the research and clinical domains. For example, automated annotation of pathology synopses with semantic labels would provide a clinical diagnostic support tool by unlocking the semantics for less experienced interpreters, and a means for knowledge mining by searching large databases of synopses for semantically similar content. Furthermore, the field of pathology is now transitioning to using digitally captured whole-slide images (WSI) for primary diagnosis (digital pathology)^4^. Scalable annotation of large WSI datasets with semantic labels from associated synopses will be essential toward developing computational pathology approaches for diagnostic support^5^.

Artificial intelligence (AI) aspires to create human-like intelligence^6^. Successful AI schemes consist largely of numerous statistical and computer science techniques collectively known as machine learning (ML)^7,8^. ML algorithms automatically extract information from data (i.e., learning, or knowledge acquisition) and then use this knowledge to make generalizations about the world^8^. Some notable examples of successful applications of ML include classifying and analyzing digital images^9^ and extracting meaning from natural language (natural language processing, NLP)^10^. One particular type of ML, called deep learning (DL), has been extremely successful in many of these tasks, particularly in image and language analysis^11^. DL algorithms are roughly modeled after the neural structure of the human brain, learning automatically to make representations from data as a hierarchy of concepts from simple to more complex ^11^, a pyramidal multi-resolution approach that should not be foreign to any pathologist. Activation weights within the different layers of the network can be adjusted according to input data, and then used to approximate a function that predicts outputs on new, unseen data^11^. The information extracted from data by DL can be represented as a set of real numbers known as “features”; within a neural network, low-dimensional embeddings of features are created to represent information as feature vectors^11^. The feature vectors produced by DL can then be used for a wide array of downstream applications, including image analysis and numerous NLP tasks such as language translation^9,12–14^.

Recently, a DL model called a *transformer* has emerged at the forefront of the NLP field^15^. Compared to previous DL-based NLP methods that mainly relied on gated recurrent neural networks with added attention mechanisms, transformers rely exclusively on attention and avoid a recurrent structure to learn language embeddings^15^. In doing so, transformers process sentences or short text holistically, learning the syntactic relationship between words through multi-headed attention mechanisms and positional word embeddings^15^. Consequently, they have shown high success in the fields of machine translation and language modeling^15,16^. Specifically, Google recently introduced Bidirectional Encoded Representations of Transformers (BERT), a transformer architecture that serves as an English language model trained on a corpus of over 800 million words in the general domain^13^. BERT encodes bidirectional representations of text using self-supervision, allowing for rich embeddings that capture meaning in human language (i.e., syntax and semantics). A classification (CLS) feature vector is an output from the last layer of the BERT model representing the embedding that captures syntactic and semantic information from the input text, which can be used to train additional ML models such as a classifier^13^. Importantly, BERT can be easily adapted to new domains by transfer learning with minimal fine-tuning, providing an ideal language model for specialized domains such as medicine^13,17,18^.

In the pathology domain, NLP methods have mainly consisted of handcrafted rule-based approaches to extract information from reports or synopses, followed by traditional ML methods such as decision trees for downstream classification ^19–23^. Several groups have recently applied DL approaches to analyzing pathology synopses, which have focused on keyword extraction versus generation of semantic embeddings^24–27^. These approaches also required manual annotation of large numbers of pathology synopses by expert pathologists for supervised learning, limiting scalability and generalization^28^.

The requirement for large-scale annotation has been a key obstacle to the supervised training of DL models in specialized domains such as pathology, given the task’s tediousness and the lack of experts with domain-specific knowledge to sufficiently label training data^29^. One approach to help mitigate this problem is known as *active learning*, where *specific* instead of random samples, samples that are underrepresented or represent weaknesses in model performance are queried and labeled as the training data^30^. In this way, a relatively small amount of labeled training data can be generalized to reach a given level of accuracy and scaled to large unlabeled datasets^30–32^. The ideal NLP approach for analyzing pathology synopses would both automatically extract features (i.e., require no manual feature engineering), generate embeddings that capture the inherent rich, semantic information, and be rapidly trainable and generalizable using a relatively small amount of expert-labeled data.

**Fig. 1:**
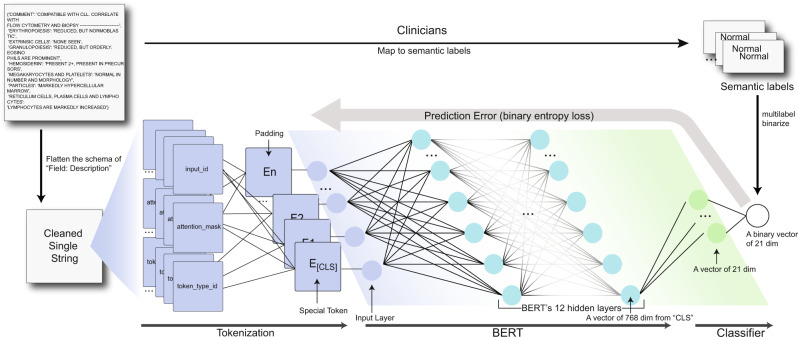
Generation of semantic labels for bone marrow aspirate synopses and modeling process. An expert reader (a clinical hematologist) interprets semi-structured bone marrow aspirate synopses and maps their contents to one or more semantic labels, which impact clinical decision-making. In order to train a model to assign semantic labels to bone marrow aspirate synopses, a synopsis first becomes a single text string and then tokenized as an input vector. The input vector will go through BERT and the classifier. The final output is a vector of size 21 (the number of semantic labels in our study). It is then compared with the ground truth vector to adjust the network weights.

In hematopathology, a bone marrow study is the foundation of making a hematological diagnosis, and consists of both a solid tissue histopathology component, called a trephine core biopsy, and a liquid cytology component, called an aspirate. As per *International Council for Standardization in Hematology* standards, an aspirate synopsis presents the morphological information in the specimen extracted by a hematopathologist in a field:description format. Each field contains a semantic summary of the pathologist’s visual interpretation of key elements of a bone marrow specimen, such as adequacy, cellularity, and the status of each hematopoietic cell lineage^33^. These synopses must then be interpreted by an expert end-reader such as a hematologist, who extracts the semantic information and then maps this to one or more core semantic labels, either “normal”, or one of various “abnormal” labels (Fig. 1 and Table 1). These conceptual labels may rarely represent a specific diagnosis; more commonly, they represent broad diagnostic categories or descriptive morphological findings^34^. The hematologist must then integrate these core semantic labels with bone marrow histopathology, ancillary testing, and clinical findings to decide on the most appropriate differential diagnosis and next steps. Often, these semantic labels do not appear in the synopsis; for example, the hematologist may map the content to the semantic label of “normal” based upon their own interpretation, but the word normal may not appear in the synopses. Therefore, bone marrow aspirate synopses form the ideal basis for evaluating NLP tools to extract embeddings that capture morphological semantics.

**Table 1:** The evolution of the semantic labels.

Iteration	New labels	Label count	Sample count
1	Acute lymphoblastic leukemia, acute myeloid leukemia, inadequate, lymphoproliferative disorder, mastocytosis, metastatic, myelodysplastic syndrome, myeloproliferative neoplasm, normal, plasma cell neoplasm	10	50
2	Erythroid hyperplasia, iron deficiency	12	83
3	Acute leukemia, acute promyelocytic leukemia, chronic myeloid leukemia, hemophagocytosis, hypercellular, hypocellular	18	
4	Basophilia, eosinophilia	20	282
5		20	296
6	Granulocytic hyperplasia	21	344
7		21	393
8		21	408
9		21	500

In each iteration, new cases and/or new labels are added to the dataset. In some iterations, we reviewed the labeled cases and added new labels to the previous cases, or added a small number of new semantic labels.

Accordingly, here we employ a BERT-based NLP model to automatically extract features and generate low-dimensional embeddings from bone marrow aspirate pathology synopses. We then apply a simple single-layer neural network classifier mapping these embeddings to one or more semantic labels *as hematopathologists*. We approach this problem as a multi-label classification using a binary relevance (BR) method, where multiple semantic labels are turned into multiple binary predictions. The model performs well in label prediction (micro-average F1 score of 0.779 ± 0.025, 0.778 ± 0.034 when evaluated by expert hematopathologists^35^). Using dimensionality reduction, chord diagrams, and a word-knockout approach, we show that the model’s embeddings capture diagnostically relevant semantic information from pathology synopses. Importantly, our model was trained using <5% of our starting dataset of over 11,000 pathology synopses using an *active learning approach*, with minimal manual data annotation by expert pathologists. Our model^36^ provides an efficient, scalable and generalizable scheme to unlock the semantic information from pathology synopses with relatively little data annotation by pathologists. We see the high relevance of our model and approach to knowledge mining, improved diagnostics and biodiscovery. A schematic illustration of our overall modeling pathway is shown in Fig. 1.

## Methods

### Pathology synopses data and preprocessing

Our study was approved by the Hamilton Integrated Research Ethics Board, study protocol 7766-C. As this study was a retrospective chart review, it was approved by the REB with waiver of consent. We collected 11,418 historical synopses for bone marrow specimens spanning April 2001 to December 2019. The original text data were saved in a spreadsheet file. Due to the format’s limitation, the synopsis structure was lost and fields were mixed with descriptions. In addition, noise (i.e., irrelevant information) including signatures from doctors and the reporting system’s context were included in the text. Here, we used our Python program^36^ to *remove the signatures, remove inline space, remove end space, and remove the reporting system*. The reduction of text noise likely helped the model learn the semantic information in this dataset more effectively. It also became more ordered and comfortable for experts to read and label these samples.

### Active learning

Only the primary dataset with 50 cases was randomly sampled, which was used to train the first model. The model then predicted the labels of the remaining 11,000 unlabeled cases. We randomly sampled *Threshold* − *Num*(*label*) cases from each rare label group based on the model’s predictions. These CRL candidates were checked by hematopathologists and had their labels verified. They were then integrated with the existing dataset to create a new dataset. A new model was then trained on this new dataset. We repeated the process until all the labels had more cases than the threshold number. We heuristically set the threshold as 20, which means that labels having less than 20 samples were considered rare labels. In the early iterations (iteration 1–5), the threshold was lowered to 10 and 15 to enrich fewer cases so that the hematopathologist would not be overwhelmed by the labeling. Iterations consisted of adding new labels and/or editing the previous labels (Table 1). As a result, the number of new labels varied in each iteration and we did not set a fixed number for how many samples the dataset was enriched by in each iteration (Algorithm 1).

If we had still found new semantic labels or the hematopathologists had thought the identified semantic labels could not cover most cases’ semantic information based on their experience, we would raise the threshold and sample more cases. We did not discover new semantic labels during the last three iterations (Table 1), and our hematopathologists confirmed the labels have covered the semantic information of most cases, which suggested the labeling is enough and CRL sampling had achieved its goals.

#### Algorithm 1: Active learning process

**Result:** A balanced dataset with more than 20 cases for each label

dataset = {50 randomly sampled cases};

**while** *COUNT*(*rareLabels*) > 0, *where* *rareLabels* = {*label*: *COUNT*(*Case*_*label*_) < 20} **do**

  Sampling process; // see Algorithm 2;

  **while** *COUNT*(*candidates*) > 100 **do**

   *threshold* = *threshold* − 5;

   Sampling process; // see Algorithm 2);

  **end**

  pathologists verify CRL candidates’ labels and may add new labels;

  dataset = dataset ∪ verified CRL;

**end**

#### Algorithm 2: Sampling process

**Result:** CRL candidates

candidates $$\leftarrow {{\emptyset}}$$;

**for** *label* *in* *rareLabels* **do**

   randomly sample *threshold* − *COUNT*(*existedCases*) CRL candidates from predicted *label* group;

   candidates.append(CRL candidates)

**end**

return candidates;

### Model training

Our overall process can be regarded as a multi-label classification, a type of supervised learning problem where an instance may be associated with multiple labels. This is different from the traditional task of single-label classification (i.e., multi-class or binary), where each sample is associated only with a single class label^37^. We approach this classification by *problem transformation*, which transforms the multi-label problem into one or more single-label classification problems. We used the most common problem transformation method, namely the BR method^38^, to transform the multi-label prediction into multiple single binary predictions. As a result, each case’s semantic label was converted into a binary vector of size 21, the number of different individual labels, to frame the training as multiple binary predictions.

Sentences in descriptions were combined into a single text string using our augmentation methods. The text was tokenized to form an input vector, which was the concatenation of “input IDs”, “attention mask”, and “token type IDs”. The input IDs were the numerical representations of words building the text; the attention mask was used to batch texts together; and token type IDs provided the classifier token *[CLS]*.

The input vector went through BERT’s 12 encoder layers. Each layer applied self-attention and passed its results through a feed-forward network to the next encoder. The output from the special *[CLS]* token was used as the input for a classifier. The classifier consisted of a dropout layer with a 0.5 dropout rate to improve the generalization and a fully connected layer with 21 nodes. It took a vector of size 768 from *[CLS]* as input and computed a logit of size 21 as output. In prediction, the *sigmoid* function (Eq. 1)^39^, turned the logit into a prediction score vector from 0 to 1:

1 $$S(x)=\frac{1}{1+{e}^{-x}}=\frac{{e}^{x}}{{e}^{x}+1}$$

The final output was a vector of size 21. The output denoted the model’s confidence that one predicted label is true. We treat each label independently and use *binary cross entropy* (Eq. 2) to calculate the loss, where *N* is the batch size and *σ* is *Sigmoid*:

2 $${\mathcal{L}}(x,y)	= \, {\rm{mean}}(L),L={\{{l}_{1},{l}_{2},\cdots ,{l}_{N}\}}^{\top },{l}_{n} \\ 	= \, -{w}_{n}[{y}_{n}{\mathrm{log}}\,\sigma ({x}_{n})+(1-{y}_{n}){\mathrm{log}}\,(1-\sigma ({x}_{n}))]$$

With the loss value, we used the Adam algorithm with weight decay fix^40^ (weight decay = 1e−2, learning rate = 1e−3) to fine-tune the network weights interconnecting the layers (Fig. 1), using HuggingFace’s Transformers^41^, a Python package. The labeled case set was randomly split into a training set (80%) and a validation set (20%). We trained models based on a training set with the ten epochs. We saved the model each epoch and compared them by the micro-average F1 score on the validation set. The best-performing model was later used to predict the labels. During the active learning stage, to make sure the training set included *all* labels, so that model could learn all the labels and help sampling CRL, we first assigned at least 1 case for each label to the training set, then randomly separated the rest to the training set and validation set to achieve the 8/2 split. After the active learning stage, we used modified Monte Carlo cross-validation (MCCV) (Algorithm 3)^42^, which was adapted by us to guarantee the validation set has at least a certain number of cases for each label, to create four final datasets. We trained four final models from them. Experts reviewed the predictions whereas the embeddings are from one randomly selected final model.

#### Algorithm 3: The adapted MCCV process

**Data:** cases, validationSizeRatio

**Result:** trainingSet, validationSet

trainSet $$\leftarrow {{\emptyset}}$$;

validationSet $$\leftarrow {{\emptyset}}$$;

tmpSet $$\leftarrow {{\emptyset}}$$;

validationSize = len(cases) * validationSizeRatio;

minValidationCaseNum = min(*COUNT*(*Case*_*label*_)) * validationSizeRatio;

random.shuffle(cases);

**for** *case in cases* **do**

  **if** *any(COUNT*(*validationCase*_*thisCaseLabel*_ < *minValidationCaseNum*) **then**

     validationSet.add(case);

  **else**

     tmpSet.add(case);

  **end**

**end**

random.shuffle(tmpSet);

**for** *case* *in* *tmpSet* **do**

  **if** *len*(*validationSet*) < *validationSize* **then**

    validationSet.add(case);

  **else**

    trainSet.add(case);

  **end**

**end**

return trainSet, validationSet

### Synopsis conversion and augmentation

The semi-structured synopses needed to be converted into single text instances first. As the schema of synopses was a table with field:description and table columns’ order would not influence its content, we could construct the text using different orders of the synopses’ parts, i.e., columns (Supplementary Fig. S1 and Supplementary Table S1).

In the computer vision field, data augmentation, a technique to increase the diversity of the training set by applying transformations such as image rotation, is usually used to solve data insufficiency challenges^43^. These transformations introduce changes but keep the data’s core patterns, and therefore, act as regularizers to reduce overfitting when training a model^44^. Likewise, thanks to the irrelevance of text order in the synopses to its semantic content, we could randomly shuffle the sequence of the synopses’ components to make different text strings to *augment* the dataset. This augmentation could also be applied for prediction (Supplementary Fig. S2). We shuffled the fields with their descriptions to create different text representations. The model computed the prediction scores on all of them. By concatenating them and only considering the maximum value for each label’s score, we obtained the result of an augmented prediction.

### Evaluation

We reviewed the NLP system’s performance in predicting labels using precision and sensitivity measures^45^. We recorded specificity, accuracy, and F1-score values based on the counts of true positives (hits), false positives (false hits), true negatives (correct rejections), and false negatives (misses) for each prediction. These performance measures were a set of equations defined as follows:

- Precision (reproducibility, PPV)
  $${\rm{precision}}=\frac{\rm{TP}}{\rm{TP+FP}}$$
- Sensitivity (recall or hit rate)
  $${\rm{recall}}=\frac{\rm{TP}}{\rm{TP+FN}}$$
- F-score (harmonic mean of precision and sensitivity)
  $${F}_{1}=2\times \frac{\rm{precision\times recall}}{\rm{precision+recall}}$$

We used micro-average F1-score, i.e., the F1-score of all labels’ aggregated contributions, to represent the overall performance. Micro-averaging emphasizes the common labels of the dataset because it puts the same importance on each sample. This was suitable for our problem, as labels that were very uncommon in the dataset were not intended to notably affect the overall F1-score if the model performed well in the other, more common labels. Micro-average F1-score^46^ is defined as:

$$	{\rm{Micro}}-{\rm{precision}}=\, \frac{{{\rm{TP}}_{\rm{sum}}}}{{{\rm{TP}}_{\rm{sum}}\,+\,{\rm{FP}}_{{\rm{sum}}}}}\\ 	 {\rm{Micro}}-{\rm{recall}}=\, \frac{{{\rm{TP}}_{\rm{sum}}}}{{{\rm{TP}}_{\rm{sum}}\,+\,{\rm{FN}}_{\rm{sum}}}}\\ 	 {\rm{Micro}}-{\rm{F}}_{1}=\, 2\times \frac{{{\rm{Micro}}\,-\,{\rm{precision}}\,\times\, {\rm{Micro}}\,-\,{\rm{recall}}}}{{{\rm{Micro}}\,-\,{\rm{precision}}\,+\,{\rm{Micro}}-{\rm{recall}}}}$$

### Word knockout

We removed a word from a synopsis and use the model to predict each label’s score. We compared the outputs with the original outputs. Since other factors remained unaltered, the change in the output was caused by the word only. We call the change the “influence score” (INF) (Supplementary Fig. S3). We did the same computation for all the words in the 500 labeled synopses’ descriptions. We grouped the influence scores by the synopses’ semantic labels and calculated their sum. Then we normalized each word’s influence score by dividing the sums with the their *L*_2_-norm (Eq. (3)) where Λ_*x*_ = {INF : label/word = *x*}.

3 $$	\, {\rm{NormINF}}{(word{X}_{labelY})}=\\ 	\, \frac{{\mathop{\sum}\limits_{{{\rm{INF}}\in \Lambda_{X}}}{\rm{INF}}}}{{\sqrt{{{\left(\mathop{\sum}\limits_{{{\rm{INF}}\in \Lambda_{A}}}{\rm{INF}}\right)}^{2}+\cdots +{\left(\mathop{\sum}\limits_{{{\rm{INF}}\in \Lambda_{Z}}}{\rm{INF}}\right)}^{2}}}}},{\left({\rm{INF}}\in {{{\Lambda }}}_{Y}\right)}$$

### Replication and blinding

This study’s procedure is programmed as a pipeline in our supplied software. The process was repeated four times on the same local servers to ensure repeatability. It was also partly run once on the Google Colab to ensure hardware independence. We also provide a Jupyter Notebook “demo_BERT_active_learning.ipynb” in our supplied software to guide other researchers to replicate our study.

Blinding is not relevant as all data were de-identified, and the study design did not entail a blinding step in the design. Researchers trained ML models to predict diagnostic labels, and hematopathologists reviewed model performance on predicting diagnostic labels. Pathologists were not aware of original diagnostic labels when evaluating model performance.

### Reporting summary

Further information on research design is available in the Nature Research Reporting Summary linked to this article.

## Results

### Using active learning to develop a labeled dataset capturing semantic information in aspirate synopses

**Fig. 2:**
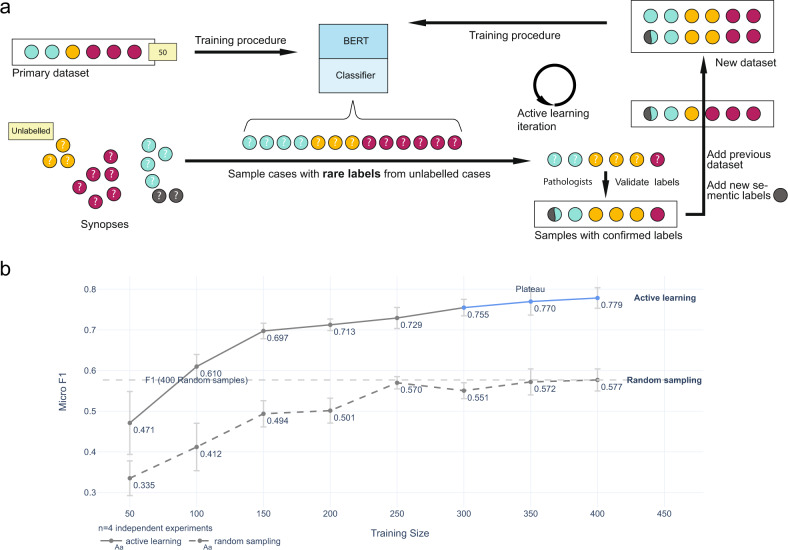
The active learning process and its result. **a** Active learning iteration for dataset building is shown. The primary purpose of iterations was to *explore* the dataset and develop semantic labels to represent the information in the 11,418 cases. In each iteration, the model trained from current dataset predicts the semantic labels for the unlabeled cases. Next, several cases are sampled from each label using an active learning approach to enrich for rare labels, to a minimum of 20 cases (a limit heuristically set) per label. Then, hematopathologists review the samples and confirm their labels. Meanwhile, new semantic labels may be discovered and they will be next iteration’s focus (they are the rarest now). These cases are merged with the current labeled dataset to form a new dataset. A new model is trained on this new dataset, and the iterations continue until the dataset includes at least 20 cases for each label. **b** Stable model performance was attained with a relatively small labeled dataset through active learning. We used the final *training dataset* of 400 labeled synopses to train models and measured their performance using the *same* 100 validation cases as a benchmark. The micro-average F1 score reaches a *plateau*, 0.770, at around 350 cases. With the same size of training data, models trained on random sampling instead of active learning can only reach a micro-average F1 score of 0.577. We have used error bars to show the standard error of the mean computed across four different experiment runs.

We first sought to develop a set of labels for the >11,000 bone marrow aspirate synopses (the raw data), with a corresponding dataset of a relatively small number of labeled synopses (the development dataset) capturing the morphological semantics in the raw data. To accomplish this efficiently, we designed an iterative *active learning* process (Fig. 2a). In this process, we used models to sample *cases with rare or underrepresented labels* (CRL) (Section “Active learning”) to help expert hematopathologists develop labels and assign these labels to new cases. Initially, a core set of labels were created by hematopathologists to represent the morphological semantics in the raw data (Table 1). Subsequently, we performed sampling-training-sampling iterations for CRL, and the number of semantic labels evolved within the process (Fig. 2a and Table 1). Each label was considered independently of all other labels; except in the case of "iron deficiency”, a “normal” label was always assigned mutually exclusive of an “abnormal” label. A given synopsis could have as many abnormal labels as the hematopathologists found necessary (Table 1). We found the number of semantic labels stabilized at 21 over seven active learning sampling-training-sampling iterations (Table 1), at which point when no new labels were deemed needed to represent the semantics of newly sampled CRL in the subsequent iterations. Over the active learning iterations, reviewed CRL were added into the development dataset until no more CRL were identified (Section “Active learning”). The final development dataset consisted of <5% of the raw data, having 500 aspirate synopses annotated with 21 different semantic labels assigned by expert hematopathologists (Table 1). We then partitioned this development dataset into 400 training cases (called the training set), and 100 validation cases (called the validation set) used to test model performance. Another 1000 cases were randomly sampled from the rest 10,918 cases (11,418 cases to 500 cases labeled) and used as an evaluation set (Supplementary Fig. S4).

To confirm the development dataset had enough cases to capture salient semantic information in the raw data, we explicitly evaluated the relationship between model performance and sample size. Here, we trained models in batches of 50 annotated synopses from the training set and used the validation set as the standard benchmark (Fig. 2b). We found that at 350 total annotated synopses, the models’ micro-average F1 score in predicting semantic labels plateaued at 0.77, suggesting that model performance stabilized, i.e., the relatively small training set covered most of the semantic information in the raw data. Furthermore, for comparison, we also performed the same experiment to train models on random samples (400 cases from the evaluation set reviewed by two expert hematopathologists who did not participate in labeling). In this case, the model only reached a micro-average F1 score of 0.62, highlighting the active learning process’s high efficiency versus random sampling(Fig. 2b). We subsequently applied the model trained on the 400 annotated training samples to extract low-dimensional BERT embeddings and map these embeddings to the semantic labels.

### Visualizing BERT-generated embeddings in the development dataset

**Fig. 3:**
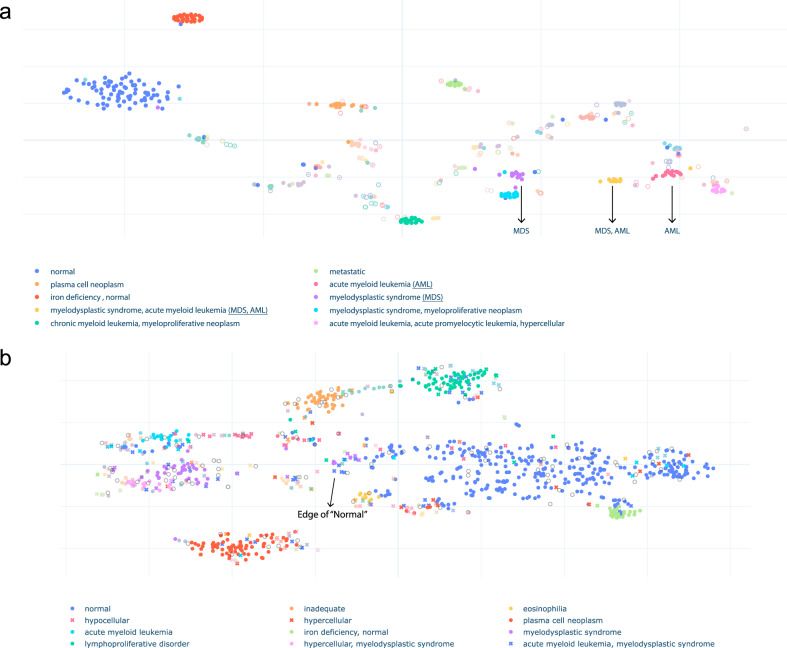
Model performance in embedding extraction. **a** 2D projection of synopsis embeddings from the 500 cases in the development set is shown. Embeddings are colored according to the combination of semantic labels. Only semantic labels with more than 12 cases are shown in the legend. The cases with the same combination of labels tend to cluster together, which suggests that the embeddings are diagnostically meaningful. Interestingly, groups with overlapping labels cluster in close proximity. For example, the “acute myeloid leukemia, myelodysplastic syndrome” group lies between the “myelodysplastic syndrome” group and the “acute myeloid leukemia” group. This suggests the model learned subtle patterns from the dataset and can map synopses to low-dimensional vectors according to diagnostic semantics. **b** 2D projection of synopsis embeddings from the 1000 cases in the evaluation set is shown. Embeddings are colored according to the combination of semantic labels. Dots represent the cases whose predictions match pathologists’ assessments. Crosses represent the cases whose predictions do not match their assessments. Only combinations with more than 12 cases are shown in the legend, and the symbol is a circle if at least 50% of cases in the group show matching between model prediction and hematopathologist review. A match means the predicted labels are the same as pathologists' expert judgment. Open-circles are the cases that were not reviewed by pathologists. [Readers can also interact with the graphs on https://storage.googleapis.com/pathopatho/label_tsne.html and https://storage.googleapis.com/pathopatho/unlabel_tsne.html, respectively]

To gain insight into diagnostic relevance of the low-dimensional embeddings (768 dimensions) generated by BERT during the active learning process, we visualized the embeddings of development dataset in 2 dimensions using t-distributed stochastic neighbor embedding (t-SNE)^47^ (Fig. 3a). We found that the embeddings tended to cluster meaningfully according to the semantic labels assigned in the development phase, suggesting a similar semantic embedding space. For example, the embeddings from synopses labeled as “normal” clustered relatively loosely, which is expected as these represent a heterogeneous group of patients. Similarly, the embeddings from synopses labeled with disease states, such as “plasma cell neoplasm” or “acute myeloid leukemia (AML)”, cluster relatively compactly, suggesting a more homogeneous clinical group as expected. Embeddings annotated more complexly with multiple labels tended to fall between major clusters; for example, the embedding labeled with “acute leukemia; myelodysplastic syndrome” fell intermediate between the clusters representing embedding for “acute leukemia” and “myelodysplastic syndrome”. These synopses represent AML with myelodysplasia-related changes (AML-MRC), which would be conceptually expected by a hematopathologist or hematologist to have features of both semantic labels^48^. These findings suggested both that the semantic labels assigned by hematopathologists were valid, and furthermore that the embeddings generated by BERT during the development phase with active learning were diagnostically relevant and captured the morphological semantics from pathology synopses.

To further evaluate our model’s ability to generate diagnostically relevant semantic embeddings, we again applied t-SNE to visualize the embeddings from an evaluation set of 1000 cases and had expert pathologists review the semantic labels (Fig. 3b). Similar to the hematopathologist-annotated development set, embeddings generated by our model from the evaluation set tended to cluster meaningfully, according to semantic labels assigned by the model (Fig. 3b). Expert hematopathologists then validated all of the labels assigned by the model to these embeddings (Fig. 3b, “closed circles”). Cases that were discrepant between the model’s prediction and the hematopathologist’s evaluation tended to have more complex label assignment (two or more semantic labels), and fall toward the edges of the clusters, suggesting these were borderline cases (Fig. 3b, “x’s”). For example, some cases predicted by the model as “hypercellular” or “granulocytic hyperplasia” were annotated as “normal” by a pathologist, which is expected given the nuances in semantic interpretation of normal by individual pathologists. Other cases demonstrated clearly discrepant model and pathologist semantic label prediction, particularly in cases with more complex labeling patterns or more broad labels such as “hypocellular”. Overall, these findings suggested that our model efficiently generated diagnostically relevant semantic embeddings from bone marrow aspirate synopses.

### Evaluating the mapping of BERT embeddings to individual semantic labels

**Fig. 4:**
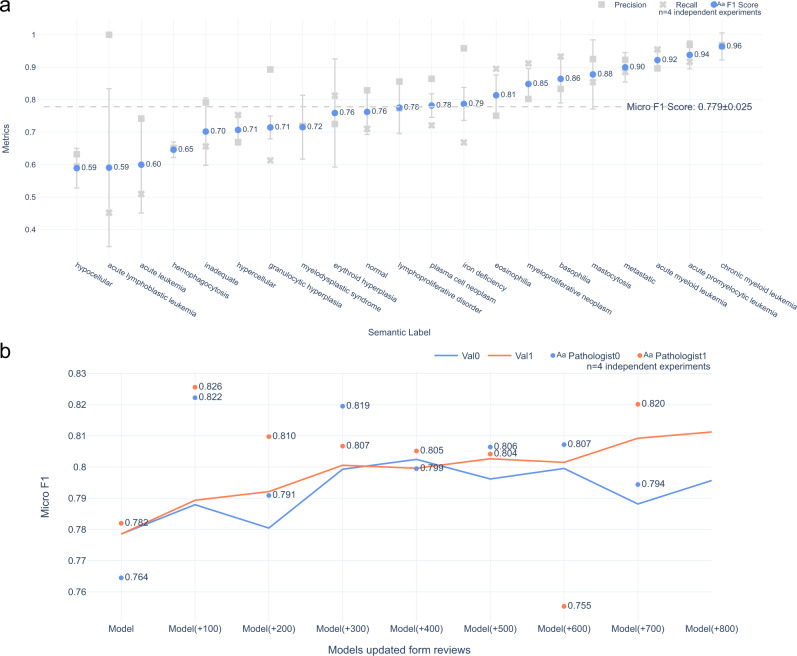
Model performance in label prediction. **a** The mean F1 scores and the standard deviation computed across four validation experiments for each label from the final models are shown. Our training strategy was to treat all labels independently. On average, the micro-average F1 score is 0.779 (Supplementary Table S2). Interestingly, the label “acute lymphoblastic leukemia” and “acute leukemia” has the lowest F1 score (0.59 and 0.60), though its sample size in the dataset is similar to that of “acute promyelocytic leukemia” (0.94). This may imply the performance is not determined solely by data size; other factors also play an important role. **b** Marginal improvement with expert feedback on randomly selected cases is shown. Pathologists reviewed the model’s predictions in 8 batches (100 randomly selected cases per batch, 800 cases in total). For each batch, the newly reviewed cases were added to the training set to re-train the model, and the updated model was used to make the next batch’s predictions (Supplementary Fig. S6). Dots represent each model generation’s performance as judged by the hematopathologists. When tested against the validation set (lines), the model started at the micro-average F1 score of 0.779. With more labeled cases provided, the model’s performance improves slightly to reach a maximum of 0.811, which shows that more cases only provide marginal improvement when randomly selected (i.e., not enriched for rare labels by active learning). We used the feedback to simulate this experiment on another three models. The values here are the average of the results from the four experiments.

The overall model performance showed a micro-average F1 score of 0.783 in predicted semantic labels (Fig. 4a). When considered independently, the model tended to predict semantic labels that constituted a specific diagnosis, or more specific diagnostic category with the highest confidence (Fig. 4a). For example, the label “chronic myeloid leukemia” was predicted with a micro-average F1 score of 1.0, but the broad descriptive label “hypocellular” was predicted with an F1 score of 0.56 (Fig. 4a). Conceptually, this is not unlike the practice of an expert reader such as a hematologist, where more specific diagnostic categories are easily predicted from a synopsis, and more broad descriptive labels may be more challenging to assign. Some specific labels, however, were predicted with lower confidence; the semantic label “acute lymphoblastic leukemia (ALL)” showed an F1 score of 0.33, while “AML” showed an F1 score of 0.9, which may reflect the imbalance in the training dataset between these diagnoses (Supplementary Fig. S5). Collectively, these findings suggested that with minimal training using active learning on a relatively small number of labeled cases, a BERT Base and simple neural network classifier model efficiently generates diagnostically relevant low-dimensional embeddings that capture morphological semantics, and maps these embeddings to one or more semantic labels with on average high confidence.

Next, we used the evaluation set reviewed by two expert hematopathologists who did not participate in labeling to further test the model’s performance to investigate the effect of increasing training data using random sampling. This aims to simulate a training process where users’ feedback is not derived from *specifically selected* samples (i.e., active learning), but rather from random samples. We found that pathologists’ micro-average F1 scores for agreement with the model’s predicted semantic labels ranged from 0.80 to 0.87, close to the stable micro-average F1 score of 0.77 we observed in model training (Figs. 2b and 4b). This both suggested that semantic labels applied in the development stage were valid, and that model’s performance tends to plateau with the initial training set. To assess the impact of pathologist evaluation on model performance, we re-trained the model in batches of 100 evaluated cases selected by random sampling, and then assessed the impact on micro-average F1 score. We found that after the predictions were adjusted by evaluating pathologists, the micro-average F1 score tended to improve (Fig. 4b). However, with more labeled cases provided, the model’s performance only improved slightly to an F1 score of 0.81, This suggested that the training cases represented the majority of morphological semantics in the dataset, and selecting more cases by random sampling provides only marginal improvement, i.e. *the CRL sampling* is highly efficient.

### Evaluating the co-occurrence of semantically similar label predictions

**Fig. 5:**
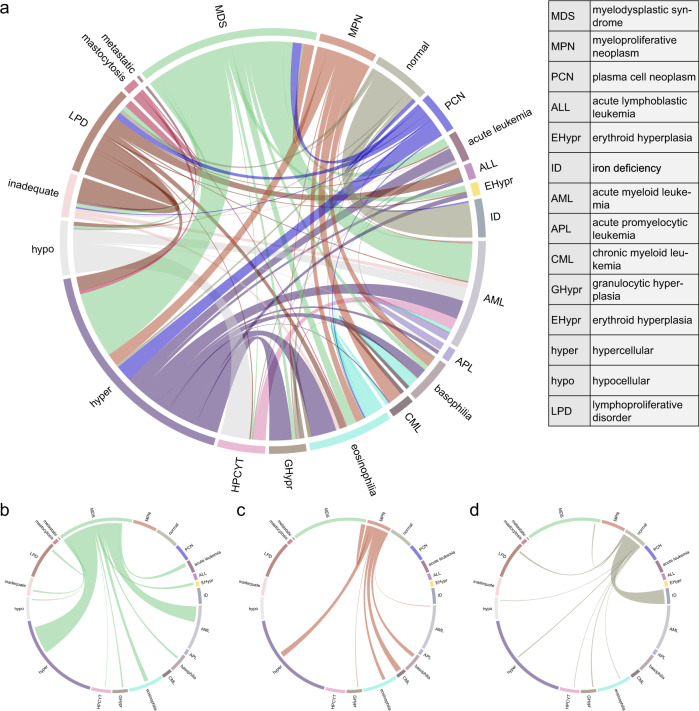
Co-occurrence of the predicted labels. **a** The chord diagram of the model predicted labels among 1000 samples is shown. Most co-occurrence relationships match semantic word relationships in hematopathology. However, the model does *not* learn the exclusiveness of the label "normal''. It may be because we treated labels independently during model training. **b** The label “myelodysplastic syndrome” co-occurred often with the labels “acute myeloid leukemia” and “hypercellular”. **c** The label “myeloproliferative neoplasm” tended to co-occur with the labels “chronic myeloid leukemia”, “hypercellular”, “basophilia”, and “eosinophilia”. **d** The model does *not* learn the exclusiveness of the label “normal”. [An interactive web version can be accessed via https://storage.googleapis.com/pathopatho/chord.html]

To further evaluate our model’s ability to capture the morphological semantics of pathology synopses, we assessed the frequency by which semantic labels predicted by our model co-occurred using a chord diagram (Fig. 5). Although our approach was a BR method^38^ where each label was considered independently, we hypothesized that if the model captured semantic information from aspirate synopses, semantically similar labels should frequently co-occur. Using the evaluation set of 1000 randomly selected synopses that were assigned semantic labels by our model, we found that semantically similar labels tended to co-occur in the model’s prediction with high frequency (Fig. 5). For example, the label “myelodysplastic syndrome” co-occurred often with the labels “acute myeloid leukemia” and “hypercellular”, as would be conceptually expected by a hematopathologist. Similarly, the label “myeloproliferative neoplasm” tended to co-occur with the labels “chronic myeloid leukemia”, “hypercellular”, “basophilia” and “eosinophilia”, again as would be conceptually expected as aspirates in myeloproliferative neoplasms often contain all of these findings. This suggested that our model captured the morphological semantics from aspirate synopses despite label prediction being a binary classification problem, allowing the model to annotate the same pathology synopsis with distinct but semantically similar labels.

### Exploring the model’s semantic label prediction process

**Fig. 6:**
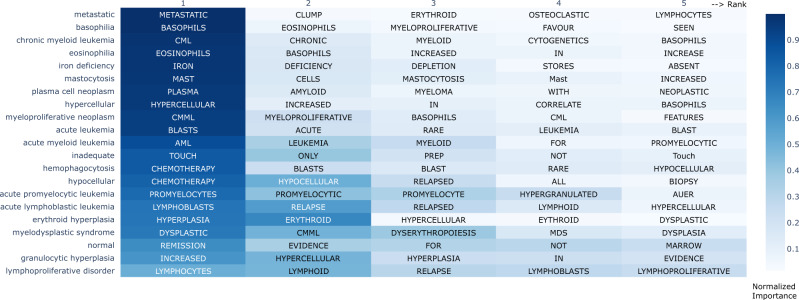
The Top-5 words the model relies on for label prediction. The color of each cell represents the *L*_2_-normalized importance score of the word. The top-1 words for the majority of labels are their acronyms or their name. For example, the top-1 influence word for "chronic myeloid leukemia'' is “CML” and the top-1 influence word for “metastatic” is “metastatic”. Furthermore, "normal'' has no words with high influence, which corresponds with clinical practice, as when specific no abnormal findings are identified by a hematopathologist, the case is semantically interpreted as normal. In this way, our knockout method provided some insight into the complex and opaque prediction process of the model.

To gain insight into how our model was assigning semantic labels, we designed and implemented a simple word-knockout approach to evaluate the influence of individual words in pathology synopses on model performance (Section “Word knockout”). With this approach, we identified the top-5 words used by the model to predict a given semantic label (Fig. 6) each associated with a normalized *importance score* (Section “Word knockout”). We found that for most semantic labels, the words weighted most highly for model prediction were either identical, or semantically similar to the label (Fig. 6, leftmost columns). For example, the semantic label “metastatic” was associated with “metastatic” or “clump”, as invasive tumor cells are often present in “clumps” in bone marrow aspirates. The semantic label “normal” was most associated with the word “remission”, as bone marrows in remission are often semantically interpreted as being normal^49^. Other words were more difficult to interpret; words like “in”, “not” and “seen” that have no obvious semantic relationship to the labels were weighted in the top 3-5 words by the model for several labels (Fig. 6). Analogous observations have been reported in other DL domains such as image recognition^50^. Overall, these findings suggested that our model learned semantically meaningful relationships between predicted labels and individual words within bone marrow aspirate synopses.

## Discussion

Tools to scalably unlock the semantic knowledge contained within pathology synopses will be essential toward improved diagnostics and biodiscovery in the era of computational pathology and precision medicine^51^. This knowledge is currently limited to a small number of domain-specific experts, forming a crucial bottleneck to the knowledge mining and large-scale diagnostic annotation of WSI that is required for digital pathology and biodiscovery. In this work, we present an NLP model based on the BERT transformer architecture and a simple neural network classifier that can automatically and efficiently generate diagnostically relevant semantic embeddings from pathology synopses, and map these embeddings to one or more labels representing semantic information. Our model accomplished this with a relatively small amount of labeled cases (training set with a size of 400), overall high confidence (micro-average F1 score of 0.779 ± 0.025, 0.778 ± 0.034 when evaluated by experts) using an iterative *active learning* process. Furthermore, we provide insight into how the model is making these predictions, which to our knowledge is the first example of exploring the mechanisms by which a transformer model generates semantic text embeddings in pathology.

We propose three main applications of our system. First, the BERT model enables the vectorization of pathology synopses. The vectorization, i.e., converting text into numerical representations in the form of feature vectors, supports many types of downstream analysis, including semantic search^52^; a database of WSI files could be queried based on a text string for WSI that contains semantically similar content. The vector distance between text or WSI is directly correlated to semantic similitude that can be analyzed using techniques such as *Euclidean distance* or *Cosine distance*: the smaller the distance, the higher the similarity. Our model, similar to Google’s universal sentence encoder^14^, could also be used to bring semantic experience into pathology research. Second, the embeddings from vectorization can be used to generate semantic labels to map patients to probable diagnostic groups. In clinical settings, pathology synopses are generally not used to facilitate computational pathology^53^. With automatic tagging, synopses can be arranged, cataloged, and retrieved in order. For example, our model could be used as a basis for a triage or workflow support tool, where synoptic reports are identified and assigned semantic labels to organize according to clinical urgency. In addition, dimensionality reduction techniques could be used to visualize embeddings providing a rapid visual assessment of probable patient diagnostic groups as a diagnostic workflow support tool. Last, combined with the word-knockout technique described in this paper, our model can compute each word’s importance score in a synopsis, highlighting essential words. Words extracted by this *knowledge mining* method can optimize the general workflow because readers generally prefer text that is easily and rapidly scannable^54^. By highlighting the most important words in synopses, end-users such as family physicians or even patients who do not have the same level of domain knowledge as a specialist may understand the synopses more effectively. Our findings also show that some words used by the model to predict semantic labels with lower confidence are not semantically similar to the label (Fig. 6). This problem is not unique to our study, as other DL domains such as image classification report similar anomalies^55^. Such findings suggest that parameters beyond individual words, such as syntactic word relationships, may be involved in model prediction. Future works may explore this in more detail. The observation that some labels were predicted with lower confidence than other labels is not unexpected in real-world datasets. Labels with lower F1 scores tend to have a lower frequency in the training set (Supplementary Fig. S5), which may partially explain this observation. One example is the semantic label ALL. Given the relatively rarity of both B and T ALL in adults, these diagnostic categories were considered as one label for this study, which may have affected model performance. Future work on larger datasets could address these rare diagnostic categories independently, specifically if designed for clinical implementation. Other factors may provide nuances in how synopses are associated with these semantic labels are structured; for example, labels such as “hypocellular” and “inadequate” tend to occur in a wide range of clinical scenarios, which may pose challenges to the model in recognizing these labels as distinct in a variety of circumstances. In addition to improving model performance with additional labeled data via active learning iterations, one solution to such problems in complex cases is “human-centric AI”, where labels assigned below certain confidence would be channeled to an expert reader for review.

Active learning is one potential solution to improve model performance and generalize a small amount of annotated training data to large datasets where high domain-specific knowledge is required. This has been a significant problem in medical domains such as pathology. We think sampling *CRL* as *specific instances* to develop a balanced dataset, where each label reaches a given threshold, is an effective adaptation of active learning for labeling tasks requiring high domain-specific knowledge. Common active learning strategies, e.g., least confidence^56^, uncertainty sampling^57^ and etc., select data based on models’ confidence, aiming to improve the models’ performance on an *established stable* set of labels. Our study was uniquely designed around a pathology clinical workflow application, requiring an active learning strategy that allowed us to develop a label set covering the semantic information in pathology synopses, as well as address imbalance in the dataset. Like any real-world dataset, the semantic labels for pathology synopses are naturally imbalanced (for example, “normal” cases are more common than “erythroid hyperplasia” cases). Thus, our active learning strategy was specifically designed to uncover new labels and also to supply underrepresented labels with more cases to alleviate imbalance. Our strategy leverages the multi-label approach to explore a dataset and discover new labels. When pathologists verify CRL candidate labels and find new semantic labels, the sampling’s focus in the next iteration will be on the new labels, which are now the *rarest*, and more cases with the new label will be found. Visually, it’s similar to moving from a semantic group’s edge boundary to its center or another boundary with a different semantic group (Fig. 3a). Second, when we add more cases with rare labels, the class imbalance will naturally be reduced. This sampling method appears to be highly efficient, as our results show the model learned the core semantic content of the dataset from a small number of training cases via this active learning approach, and more cases randomly selected only provide marginal improvement. Additional active learning strategies, such as least confidence, uncertainty sampling, and discriminative active learning^58^, could be explored in future work once a stable and balanced set of labels is attained. One could envision using such approaches in an “adaptive AI system” where pathologists continually evaluate model performance and provide feedback in real-time based on underrepresented labels, to a point where the model performance is difficult to distinguish from an expert colleague. Such an approach may be an avenue toward validating and implementing a similar model as a clinical workflow support tool.

We used a BR method (Section “Model training”), to transform the multiple semantic labels into multiple binary predictions. The drawback of this method is that it ignores the information that can be extracted from considering label correlations; this may be why the model does not grasp the exclusiveness of “normal” (Fig. 5). However, this approach is resistant to overfitting label combinations because it does not expect samples to be related to previously observed label combinations. Therefore, it can handle very irregular labeling (some labels are exclusive and some are inclusive), which is expected in pathology domains. Moreover, since labels have a one-to-one relationship to binary models, labels can be added and removed without noticeably affecting the rest of the model. These advantages make it applicable to the annotation of pathology synopses, where the sample size is small (high risk of overfitting) and the labels are continuously evolving (Table 1). Although the number of semantic labels is 21 as active learning process concluded, this number could be increased as additional pathologists continue to review cases leading to increasingly complex and granular combinations of semantic labels.

Finally, our approach is relatively straightforward compared with other studies^19–23^ in this area. The rule-based systems need to formalize handcrafted rules for specific tasks, while our method skips the feature engineering and further manual intervention. Training a neural network from scratch requires an extensive training corpus, but by fine-tuning the pre-trained BERT model with additional augmentation steps (described in the “Methods” section), e.g., sampling-training-sampling iteration, data augmentation, and prediction augmentation, we can use this sophisticated transformer model with only 500 labeled samples and achieve 0.779 ± 0.025 micro-average F1-score during final evaluation. We have packaged our approach as a Python application. Other researchers only need to provide their samples and labels. Therefore, we expect this model will be easily generalizable and scalable to other pathology and medical domains.

## Supplementary information

Supplementary Data 1

Supplementary information

Description of Additional Supplementary Files

Reporting Summary

## Data Availability

The data that support the findings of this study are available on reasonable request from the corresponding author [C.J.V.C.], pending local REB approval. The data are not publicly available due to them containing information that could compromise research participant privacy/consent. Source data underlying the main figures in the manuscript are available as Supplementary Data 1.

## Code availability

Code for data cleaning and analysis associated with this study is available at https://zenodo.org/record/4711413. Code for the application that collects experts’ review is available at https://zenodo.org/record/4711438.
