# Supplementary material for: A BERT model generates diagnostically relevant semantic embeddings from pathology synopses with active learning
Source: Commun Med (Lond). 2021 Jul 5;1:11. doi: 10.1038/s43856-021-00008-0 (PMC9053264; doi:10.1038/s43856-021-00008-0)
Supplement: Supplementary file 2 — Supplementary information [file 43856_2021_8_MOESM2_ESM.pdf]

	Micro F1
Without Upsampling	0.746 ± 0.010
With Upsampling	0.779 ± 0.025

Table S1: **The micro-average F1 scores comparison between models trained with and without upsampling.** We also show the standard deviation computed across 4 experiments.

Label	F1 Score	Precision	Recall
chronic myeloid leukemia	0.964 ± 0.042	0.964 ± 0.071	0.969 ± 0.062
acute promyelocytic leukemia	0.938 ± 0.043	0.969 ± 0.062	0.917 ± 0.096
acute myeloid leukemia	0.922 ± 0.034	0.897 ± 0.078	0.955 ± 0.059
metastatic	0.9 ± 0.045	0.923 ± 0.09	0.885 ± 0.079
mastocytosis	0.878 ± 0.107	0.925 ± 0.15	0.854 ± 0.138
basophilia	0.864 ± 0.074	0.833 ± 0.192	0.933 ± 0.078
myeloproliferative neoplasm	0.848 ± 0.048	0.802 ± 0.098	0.912 ± 0.075
eosinophilia	0.814 ± 0.062	0.75 ± 0.087	0.895 ± 0.081
iron deficiency	0.787 ± 0.051	0.958 ± 0.083	0.668 ± 0.036
plasma cell neoplasm	0.782 ± 0.036	0.864 ± 0.094	0.721 ± 0.066
lymphoproliferative disorder	0.775 ± 0.08	0.856 ± 0.24	0.773 ± 0.158
normal	0.762 ± 0.07	0.829 ± 0.055	0.71 ± 0.097
erythroid hyperplasia	0.759 ± 0.166	0.725 ± 0.222	0.812 ± 0.142
myelodysplastic syndrome	0.715 ± 0.098	0.718 ± 0.137	0.72 ± 0.091
granulocytic hyperplasia	0.715 ± 0.035	0.893 ± 0.137	0.613 ± 0.096
hypercellular	0.707 ± 0.047	0.669 ± 0.057	0.753 ± 0.067
inadequate	0.702 ± 0.104	0.792 ± 0.25	0.656 ± 0.021
hemophagocytosis	0.646 ± 0.024	0.651 ± 0.05	0.649 ± 0.081
acute leukemia	0.6 ± 0.149	0.742 ± 0.211	0.51 ± 0.132
acute lymphoblastic leukemia	0.591 ± 0.243	1.0 ± 0.0	0.452 ± 0.254
hypocellular	0.589 ± 0.061	0.632 ± 0.156	0.594 ± 0.172

Table S2: **The F1 scores for each label from the 4 final models.** We also show the standard deviation computed across 4 validation experiments.

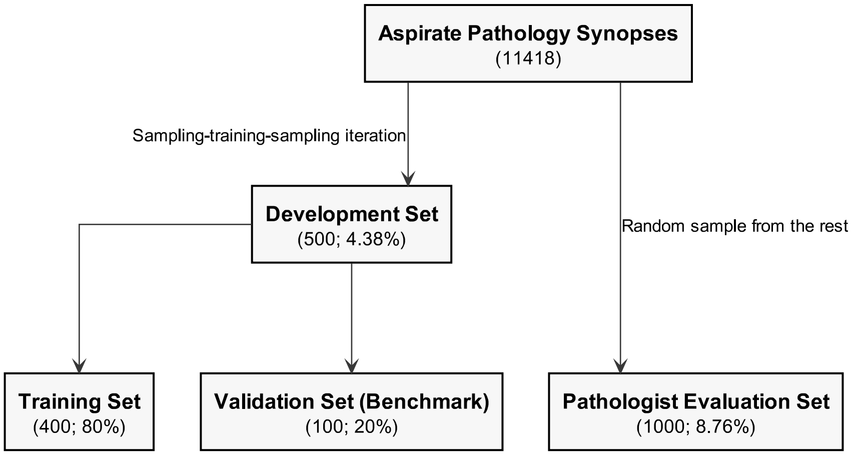

Figure S1: **A summary of the datasets.** The final development dataset consisted of < 5% of the 11,418 synopses, which were annotated with semantic labels by expert hematopathologists. A total of 400 synopses were used to train models and 100 of them were set as a benchmark to measure the model's performance. Another 1000 cases were randomly sampled for pathologists' review.

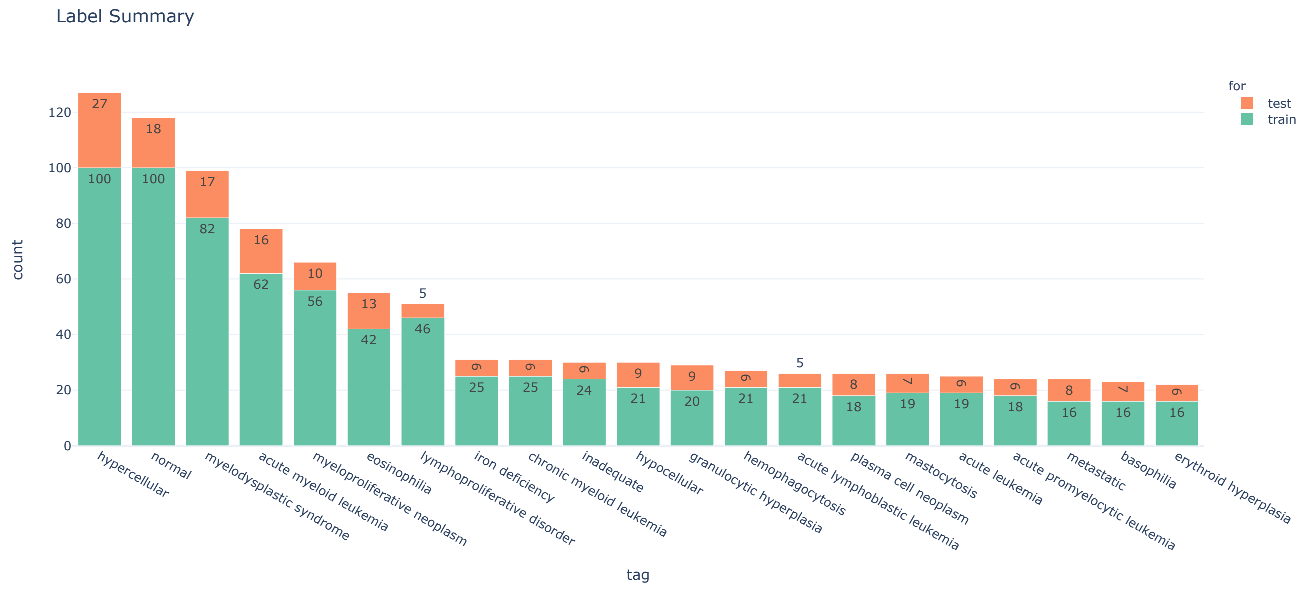

Figure S2: **The sample size for each semantic label in one of the final development datasets.** Each label had at least 20 samples in total with at least 5 samples being in the test set.

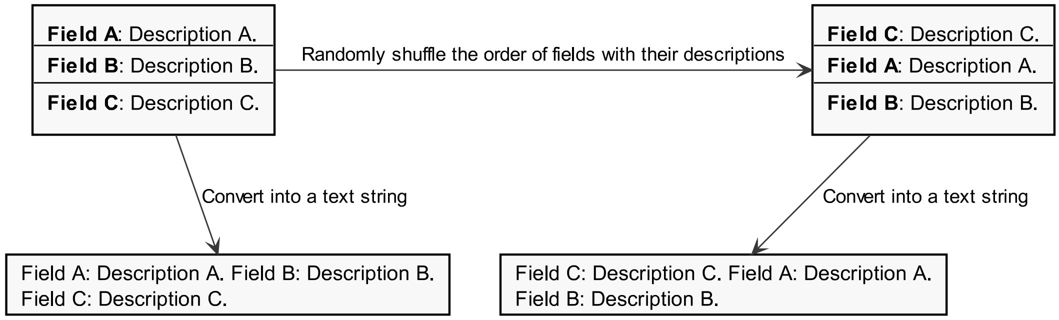

Figure S3: **The conversion from a semi-structured synopsis to a single text string.** It also shows how the sequence of the synopsis' components was shuffled to make different text representations.

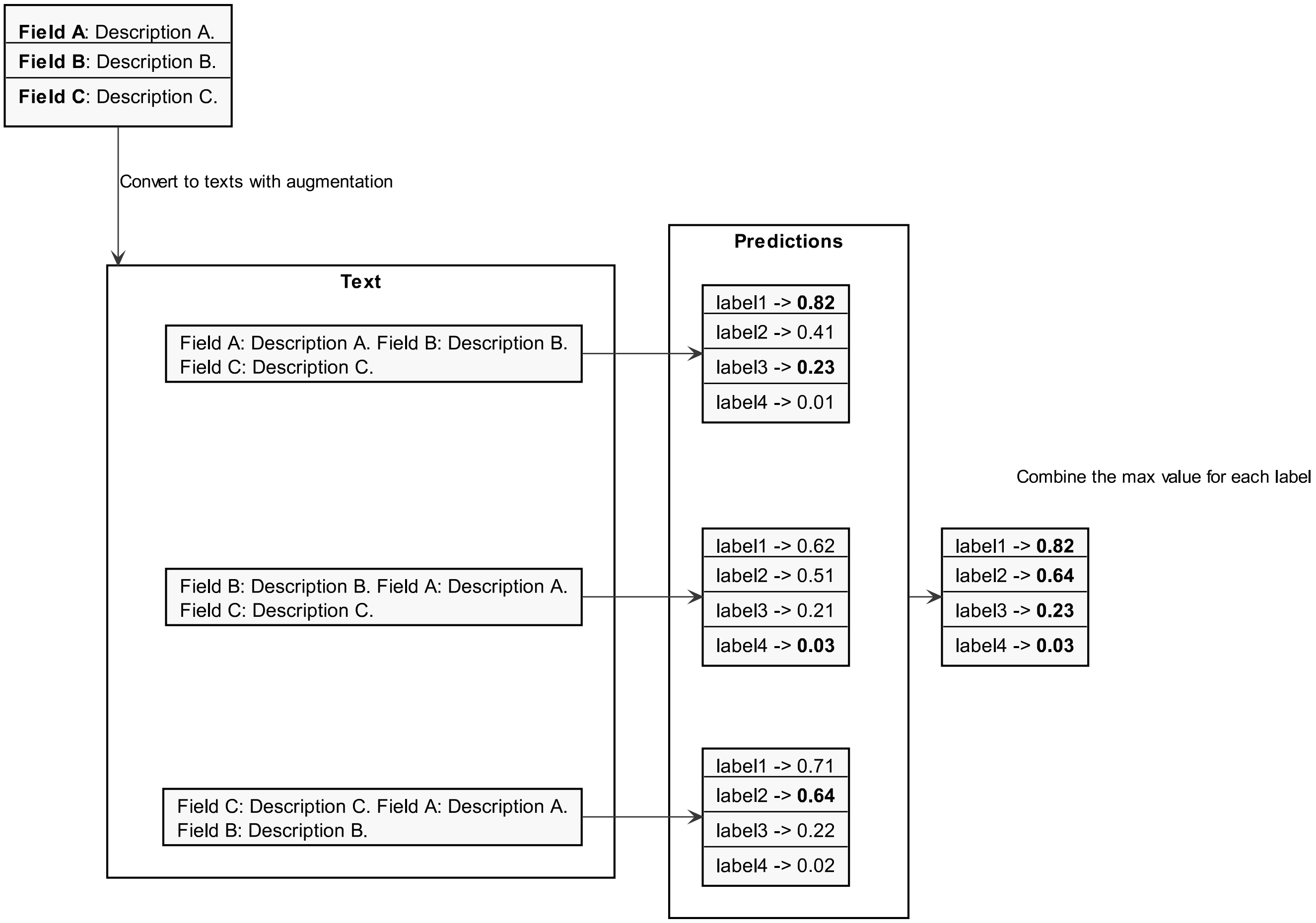

Figure S4: **The application of augmentation during prediction.** The fields and their values are shuffled to create various text representations. The model determined the prediction scores. By combining the scores and only using each label's maximum output, we obtained the result of an augmented prediction.

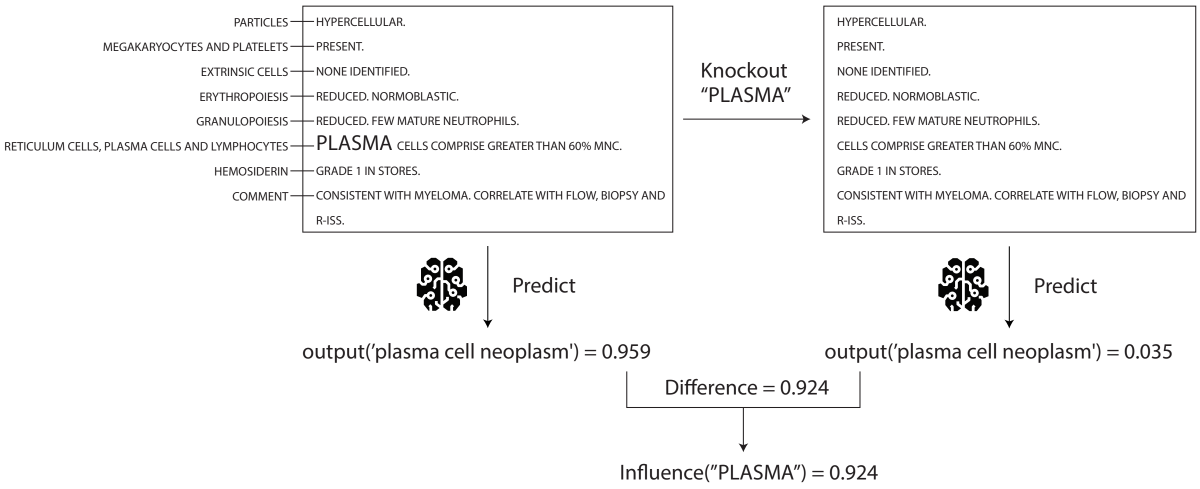

Figure S5: **The word knockout method.** The influence score of the word "PLASMA" for the label "plasma cell neoplasm" was computed as follows. We removed the word "PLASMA" from the original synopsis (left) to create a new synopsis. The model gave both synopses prediction scores for "plasma cell neoplasm". Since the other variables remained the same, the change of the prediction scores was caused solely by knocking out the word. We assigned the difference between the prediction score before and after knockout as the word's influence on the label "plasma cell neoplasm".

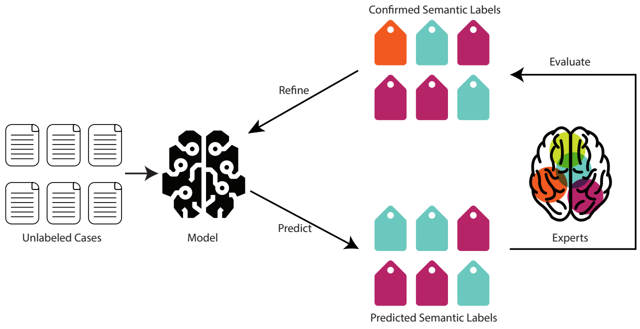

(a) **The review process.** Pathologists interacted with a desktop application to evaluate the model's performance. The evaluation results were saved into a centralized database. After accumulating a sufficient number of results, the model was further trained on these results.

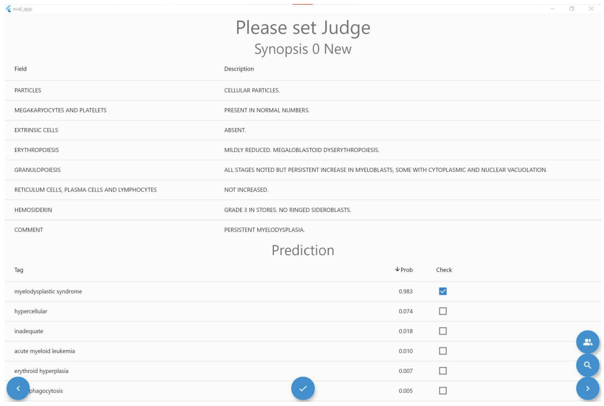

(b) The GUI of the evaluation application.

Figure S6: The final evaluation process.
