# Supplementary material for: A BERT model generates diagnostically relevant semantic embeddings from pathology synopses with active learning
Source: Commun Med (Lond). 2021 Jul 5;1:11. doi: 10.1038/s43856-021-00008-0 (PMC9053264; doi:10.1038/s43856-021-00008-0)
Supplement: Supplementary file 3 — Description of Additional Supplementary Files [file 43856_2021_8_MOESM3_ESM.pdf]

Description of Additional Supplementary Files

File Name: Supplementary Data 1

Description: Source data underlying the main figures in the manuscript are available here. “active_learning_result.csv” includes the data used to compare the effectiveness between active learning and random sampling, which is the source of *Fig. 2b*. “unlabel_tsne.csv” and “label_tsne.csv” include the 2-D projections of the cases’ 768-D embeddings, which are the source of *Fig. 3*. “dev_result.csv” includes the model performance metrics during development, which is the source of *Fig. 4a*. “review_result.csv” includes the results from experts’ review, which is the source of *Fig. 4b*. “mat_abbr.csv” includes label co-occurrence counts, which is the source of *Fig. 5*. “kws_influence.csv” includes the words’ influence scores for each label, which is the source of *Fig. 6*.
