# Supplementary material for: A BERT model generates diagnostically relevant semantic embeddings from pathology synopses with active learning
Source: Commun Med (Lond). 2021 Jul 5;1:11. doi: 10.1038/s43856-021-00008-0 (PMC9053264; doi:10.1038/s43856-021-00008-0)
Supplement: Supplementary file 4 — Reporting Summary [file 43856_2021_8_MOESM4_ESM.pdf]

nature research

Corresponding author(s): COMMSMED-21-0060-T

Last updated by author(s): May 11, 2021

Reporting Summary

Nature Research wishes to improve the reproducibility of the work that we publish. This form provides structure for consistency and transparency in reporting. For further information on Nature Research policies, see our Editorial Policies and the Editorial Policy Checklist.

Statistics

For all statistical analyses, confirm that the following items are present in the figure legend, table legend, main text, or Methods section.

n/a	Confirmed	
☐	☒	The exact sample size (n) for each experimental group/condition, given as a discrete number and unit of measurement
☐	☒	A statement on whether measurements were taken from distinct samples or whether the same sample was measured repeatedly
☒	☐	The statistical test(s) used AND whether they are one- or two-sided<br>*Only common tests should be described solely by name; describe more complex techniques in the Methods section.*
☒	☐	A description of all covariates tested
☒	☐	A description of any assumptions or corrections, such as tests of normality and adjustment for multiple comparisons
☒	☐	A full description of the statistical parameters including central tendency (e.g. means) or other basic estimates (e.g. regression coefficient) AND variation (e.g. standard deviation) or associated estimates of uncertainty (e.g. confidence intervals)
☒	☐	For null hypothesis testing, the test statistic (e.g. F, t, r) with confidence intervals, effect sizes, degrees of freedom and P value noted<br>*Give P values as exact values whenever suitable.*
☒	☐	For Bayesian analysis, information on the choice of priors and Markov chain Monte Carlo settings
☒	☐	For hierarchical and complex designs, identification of the appropriate level for tests and full reporting of outcomes
☒	☐	Estimates of effect sizes (e.g. Cohen's d, Pearson's r), indicating how they were calculated

Our web collection on statistics for biologists contains articles on many of the points above.

Software and code

Policy information about availability of computer code

Data collection	Code for the application that collects experts' review is available at https://zenodo.org/record/4711438.
Data analysis	Code for data cleaning and analysis associated with this study is available at https://zenodo.org/record/4711413.

For manuscripts utilizing custom algorithms or software that are central to the research but not yet described in published literature, software must be made available to editors and reviewers. We strongly encourage code deposition in a community repository (e.g. GitHub). See the Nature Research guidelines for submitting code & software for further information.

Data

Policy information about availability of data

All manuscripts must include a data availability statement. This statement should provide the following information, where applicable:

- Accession codes, unique identifiers, or web links for publicly available datasets
- A list of figures that have associated raw data
- A description of any restrictions on data availability

The data that support the findings of this study are available on reasonable request from the corresponding author [CJVC], pending local REB approval. The data are not publicly available due to them containing information that could compromise research participant privacy/consent.
Source data underlying the main figures in the manuscript are available as Supplementary Data at https://osf.io/pfztq/.

Field-specific reporting

Please select the one below that is the best fit for your research. If you are not sure, read the appropriate sections before making your selection.

☒ Life sciences ☐ Behavioural & social sciences ☐ Ecological, evolutionary & environmental sciences

For a reference copy of the document with all sections, see nature.com/documents/nr-reporting-summary-flat.pdf

Life sciences study design

All studies must disclose on these points even when the disclosure is negative.

Sample size	N = 11,418
Data exclusions	N/A
Replication	This study's procedure is programmed as a pipeline in our supplied software. The process has been repeated four times on the PHRI servers to ensure repeatability. It has also partly run one time on the Google Colab to ensure hardware independence. We also provide a Jupyter Notebook "demo_BERT_active_learning.ipynb" in our supplied software to guide other researchers to replicate our study.
Randomization	N/A not a randomized study design
Blinding	Blinding is not relevant as all data were de-identified, and study design did not entail a blinding step in the design. Researchers ML mod trained ML models to predict diagnostic labels, and pathologists reviewed model performance on predicting diagnostic labels. Pathologists were not aware of original diagnostic labels when evaluating model performance.

Reporting for specific materials, systems and methods

We require information from authors about some types of materials, experimental systems and methods used in many studies. Here, indicate whether each material, system or method listed is relevant to your study. If you are not sure if a list item applies to your research, read the appropriate section before selecting a response.

Materials & experimental systems

n/a	Involved in the study	
☒	☐	Antibodies
☒	☐	Eukaryotic cell lines
☒	☐	Palaeontology and archaeology
☒	☐	Animals and other organisms
☒	☐	Human research participants
☐	☒	Clinical data
☒	☐	Dual use research of concern

Methods

n/a	Involved in the study	
☒	☐	ChIP-seq
☒	☐	Flow cytometry
☒	☐	MRI-based neuroimaging

Clinical data

Policy information about clinical studies

All manuscripts should comply with the ICMJE guidelines for publication of clinical research and a completed CONSORT checklist must be included with all submissions.

Clinical trial registration	N/A
Study protocol	HiREB 7766-C
Data collection	Retrospective chart review with waiver of consent
Outcomes	N/A
